# Key Performance Indicators for Evaluation and Treatment of Early Alzheimer's Disease with Anti Amyloid Monoclonal Antibody in Neurology Community Clinics

**DOI:** 10.1002/alz70858_104139

**Published:** 2025-12-26

**Authors:** Jose Soria‐Lopez, Lena Bueno, Jannah Gaudia, Sequoia Ochoa‐Cipes, Uriel Romero, Claudia Asencio, Kevin McGehrin, Jacob Hall, Branko Huisa

**Affiliations:** ^1^ The Neuron Clinic, San Diego, CA, USA

## Abstract

**Background:**

Disease‐modifying therapies, such as anti‐amyloid monoclonal antibodies, have emerged as treatment options to slow cognitive and functional decline in early Alzheimer's Disease (AD). These therapies require careful implementation in community clinics, with considerations including patient selection, timely access to therapy, and monitoring for adverse events. Therefore, the development of Key Performance Indicators (KPIs) is crucial to ensure the effective delivery of these novel treatments and to improve outcomes in real‐world settings.

**Methods:**

We developed a clinical program for diagnosing and treating early AD within community clinics in Southern California. Patients confirmed to have early‐stage AD were offered access to disease‐modifying therapy with anti‐amyloid monoclonal antibody, administered through in‐clinic based infusion centers. Additional patients were enrolled and monitored while receiving treatment at external infusion centers. KPIs relevant to patient care were identified. Demographic and clinical data were analyzed using non‐parametric statistical tests and regression analyses to assess the utility of these KPIs in guiding clinical practice.

**Results:**

Between April 2023 and December 2024, a total of 506 patients were enrolled in the program. Among them, 108 received treatment, 140 were pending treatment, and 258 were disqualified.

Key metrics included:

**Negative AD Biomarker Rate**: Percentage of patients with negative AD specific biomarker results as the reason for disqualification of treatment.

**Treatment Rate**: Percentage of eligible patients receiving therapy, analyzed independently of insurance coverage.

**Door‐to‐Treatment Time**: Time from first clinical evaluation to first infusion treatment.

**Dropout Rate**: Proportion of patients discontinuing therapy.

**Rates of Amyloid Related Imaging Abnormalities (ARIA)**

**Conclusions:**

The evaluation and treatment of early Alzheimer's disease with anti‐amyloid monoclonal antibody therapy is both feasible and safe within community neurology clinics. Streamlined triaging, simplified care coordination, and rigorous quality and safety monitoring are critical for success. Monitoring key performance indicators—including 1) Negative AD Biomarker Rate, 2) Treatment Rate, 3) Door‐to‐Treatment time, 4) Dropout Rate, and 5) ARIA rates—facilitates the standardization and scalability of care delivery. These KPIs offer a structured approach to optimizing treatment outcomes and broadening access to disease‐modifying therapies in real‐world settings.

